# Lack in Periodontal Care of Patients Suffering from Severe Heart Diseases—Results after 12 Months Follow-Up

**DOI:** 10.3390/jcm9020352

**Published:** 2020-01-27

**Authors:** Dirk Ziebolz, Sylvia Friedrich, Christian Binner, Josephine Rast, Mirjam Eisner, Justus Wagner, Jan Schmickler, Tanja Kottmann, Rainer Haak, Michael A. Borger, Sven Lehmann, Andreas Oberbach, Jens Garbade, Gerhard Schmalz

**Affiliations:** 1 Department of Cariology, Endodontology and Periodontology, University of Leipzig, 04103 Leipzig, Germany; friedrich.sylvia@googlemail.com (S.F.); Josephine.Rast@medizin.uni-leipzig.de (J.R.); mirjamcharlotte@web.de (M.E.); justus7@gmx.de (J.W.); schmickler.jan@gmail.com (J.S.); rainer.haak@medizin.uni-leipzig.de (R.H.); Gerhard.Schmalz@medizin.uni-leipzig.de (G.S.); 2 University Department of Cardiac Surgery, Heart Center Leipzig, 04289 Leipzig, Germany; Christian.Binner@leipzig-heart.de (C.B.); Michael.Borger@helios-gesundheit.de (M.A.B.); Sven.Lehmann@medizin.uni-leipzig.de (S.L.); Jens.Garbade@medizin.uni-leipzig.de (J.G.); 3 CRO Dr. med. Kottmann GmbH & Co. KG, 59077 Hamm, Germany; tk@cro-kottmann.de; 4 Department of Diagnostics, Fraunhofer Institute for Cell Therapy and Immunology, 04103 Leipzig, Germany; andreas.oberbach@medizin.uni-leipzig.de

**Keywords:** heart transplantation, left-ventricular assist device, heart failure, oral health, dental care

## Abstract

Background: To assess whether the standardized recommendation of patients with heart failure (HF), left-ventricular assist device (LVAD) and heart transplantation (HTx) to visit their dentist leads to improved oral conditions after 12 months. Methods: Patients from the Department of Cardiothoracic Surgery, Leipzig Heart Centre, Germany were examined at baseline and after 12 months. A dental (decayed-, missing-, and filled-teeth index (DMF-T)) and periodontal examination (periodontal probing depth, clinical attachment loss) was performed. At baseline, patients received a standardized recommendation to visit their dentist. At follow-up, a standardized questionnaire regarding the dental consultation was applied. Results: Eighty-eight participants (HTx: 31, LVAD: 43, HF: 14) were included. The majority of patients (79.5%) followed the recommendation to visit their dentist. Within the total cohort, periodontal treatment need was significantly reduced from 91% (baseline) to 75% (follow-up; *p* < 0.01). Only 10% of total cohort stated that they received periodontal treatment. The outcome in periodontal and dental treatment need at follow-up appointment revealed no statistically significant associations to the questionnaire regarding dentist consultation (*p* > 0.05). Conclusions: The simple recommendation to visit the dentist appears not enough to obtain sufficient dental and periodontal conditions in patients with severe heart diseases. Thereby, a lack in periodontal treatment of patients with HF, HTx and LVAD was identified, making interdisciplinary dental special care programs recommendable.

## 1. Introduction

Cardiovascular diseases are a major global health issue. Their prevalence and incidence is high and they are responsible for about 17.5 million deaths per year worldwide [1,2]. Heart failure is an important condition, whereby its end-stage often leads to the necessity of circulatory support and/or heart transplantation [3,4,5]. Therefore, patients with heart failure, patients with circulatory support e.g., after implantation of left-ventricular assist device (LVAD) as well as after heart transplantation, are a large group of patients, which are also of relevance in dental care. Thereby, several dental considerations for these patients, especially in context of transplantation were already mentioned many years ago [6,7]. On the one hand, an early dental rehabilitation of these patients before and sufficient maintenance after transplantation is recommended to reduce the risk for infectious complications [8]. Insufficient oral health in patients with heart diseases was also reported to be a risk factor for hospitalization and mortality [9], while improved oral health and care can reduce the risk of cardiovascular events [10]. On the other hand, dental treatment of these patients is related to special characteristics, e.g., the risk for bleeding complications related to anticoagulation [11]. Accordingly, special attention in dental care of these patients is necessary.

In contrast, patients with heart transplantation were found to suffer from high periodontal treatment need [12]. While further data for heart transplant recipients are lacking, findings of patients with other solid organ transplantations suggest the absent of sufficient rehabilitation before and maintenance after transplantation [13]. This gap in dental healthcare of these patients is supported by different examinations that presented high treatment need in patients before and after organ transplantation [14,15,16,17]. In this respect, a previous study by this working group showed a high periodontal treatment need of patients with heart failure and after heart transplantation [18]. Up until now, possible solutions to improve this insufficient dental care situation remain questionable. It has been reported that oral health promotion activities may lead to better periodontal status, positive effects on systemic inflammation and endothelial function of patients with cardiovascular diseases [19]. However, data regarding this issue are still rare [19]. Thereby, the appropriate oral health intervention remains unclear. Different views on dental care between general physicians and dentists as well as a lack of sufficient interdisciplinary collaboration have been reported as a potential major problem in this context [20]. It appears unclear, if there is no allocation of the patients to the dentist, if the patients follow this allocation and if the patients receive the appropriate therapy by their dentist. Regularly, patients should be allocated to dentist, especially prior to transplantation [8]. However, it is often not checked, whether this allocation leads to an improved oral situation of these patients.

Therefore, the current study aimed to assess whether the standardized recommendation of patients with heart failure (heart failure, LVAD and heart transplantation) to visit their dentist leads to improved oral conditions after a period of 12 months. Moreover, potential reasons for the absence of an improvement in oral health and treatment need were evaluated. Based on this examination, it was aimed to detect whether the simple recommendation to visit the dentist is appropriate to reduce dental and periodontal treatment need. For the interpretation of oral conditions at baseline, a healthy comparison group was included.

## 2. Methods

This clinical investigation was designed as a prospective cohort study with a follow-up examination after 12 months. The current study was reviewed and approved by the ethics committee of the Medical Faculty of University of Leipzig (No: 414/16-ek). All included patients were informed verbally and in writing about the study and provided written informed consent for participation.

### 2.1. Patients

Patients with different severe heart diseases/related conditions who attended the Department of Cardiothoracic Surgery, Leipzig Heart Centre, Leipzig, Germany for routine follow-up appointment were recruited. Thereby, patients after heart transplantation (HTx), with left-ventricular assist device (LVAD) or heart failure (HF) were informed about the study and asked for their voluntary participation. The inclusion criteria were one of the above-mentioned disease/condition, mean age of at least 18 years as well as the ability to provide informed consent for participation. The following exclusion criteria were formulated:

- worse general health status making a clinical examination impossible;
- auto-immune diseases (e.g., rheumatoid arthritis, chronic inflammatory bowel disease);
- infectious diseases (hepatitis A, B, C, tuberculosis, HIV);
- pregnancy.

For all participants, clinical-cardiological data (e.g., systemic/heart disease, medication), smoking habits (smoker: currently smoking, former smoker: smoking within five years before examination, non-smoker: no smoking for at least five years), age and gender were extracted from the patient’s medical records.

For comparison of oral health situation of the total cohort of participants with heart diseases, a healthy comparison group (HC) was composed. The HC contained patients without any cardiac diseases attending the Department of Cariology, Endodontology and Periodontology, University of Leipzig, Germany for their routine control appointments. From the available pool of generally healthy individuals, a cohort of participants with comparable age, gender and smoking habits like the heart diseased patients was composed. Thereby, a matching with regard to age, gender and smoking was performed as good as possible. The in- and exclusion criteria were equal for both groups.

### 2.2. Oral Examination

The oral examinations were executed at the Department of Cardiothoracic Surgery, Leipzig Heart Centre, Leipzig, Germany or at the Department of Cariology, Endodontology and Periodontology, University of Leipzig, Germany, respectively. The participants with severe heart diseases received 2 g Amoxicillin as an antibiotic prophylaxis before examination [21].

Dental examination: To evaluate the dental status, the decayed- (D-T), missing- (M-T) and filled-teeth (F-T) index (DMF-T) was assessed according to WHO [22]. Carious teeth, showing a lesion with cavitation of the tooth surface were evaluated as D-T. Missing teeth, excluding third molars, were added to M-T component. In contrast to the original assessment of M-T [22], all missing teeth were recorded, irrespective of the reason for tooth loss (caries or periodontitis). Filled or crowned teeth have been assigned to the F-T component. If at least one carious lesion deserving invasive dental intervention (D-T > 0) was present, dental treatment need was rated.

Periodontal examination: At six measurement points per tooth, periodontal probing depth and clinical attachment loss was assessed with a periodontal probe (PCP 15, Hu-Friedy, Chicago, IL, USA). If periodontal probing depth ≥ 3.5mm in at least two different sextants was present, periodontal treatment need was rated according to Periodontal Screening Index (PSI) [23,24]. The presence of dental and/or periodontal treatment need was summarized as overall dental treatment need.

### 2.3. Questionnaire

At the follow-up appointment, all participants answered a standardized questionnaire. Thereby, it was assessed whether patients visited their dentist in the previous 12 months following the recommendation or because of complaints. Furthermore, therapy measures applied by the consulted dentist were evaluated. Moreover, questions were asked about antibiotic prophylaxis and information from the dentist. Additionally, patients were asked regarding dental behavior since heart disease was diagnosed (Table 1).

### 2.4. Study Flow

Prior to clinical examination, the dentists who performed the investigation underwent a calibration process. For this, all of these dentists examined the same generally healthy patients independently, until the results of the clinical oral investigation were nearly equal (kappa >0.8). The dentist who performed the follow-up examination was calibrated in the same way with the three baseline investigators. After written informed consent, patients with heart diseases were examined at the Department of Cardiothoracic Surgery, Leipzig Heart Centre, Leipzig, Germany, once by three experienced and calibrated dentists under standardized conditions. Thereby, a complete oral examination as described above was performed. Afterwards, patients obtained a standardized doctor´s letter that included the information that patients received dental examination and whether treatment need was detected. Thus, patients received the recommendation to visit their dentist and deliver the doctor´s letter to their treating dentist. Twelve months after patients received this recommendation (follow-up), all participants underwent a complete oral examination again by another experienced dentist to detect whether dental and/or periodontal situation had changed/improved. Furthermore, the questionnaire regarding their dental consultation was applied to detect whether patients visited their dentist based on the recommendation and if the dentist performed any therapy measures with regard to the detected treatment need at baseline. Only patients who completed the whole follow-up were included for analysis. At baseline, 329 patients with severe heart diseases (HTx: *n* = 112, HF: *n* = 89, LVAD: *n* = 128) were screened and examined. Only 88 patients (26.7%) completed the follow-up examination and were included in this study.

### 2.5. Statistical Analysis

The statistical analysis was performed with SPSS for Windows, version 24.0 (SPSS Inc., Chicago, IL, USA). Normal distribution was tested with the Kolmogorov–Smirnov test. The non-normal distributed samples were analyzed with the Mann–Whitney U test as a non-parametric test. Two related samples were analyzed with the Wilcoxon test. For more than two independent, non-normal distributed samples, the Kruskal–Wallis test was applied. Comparing the data between baseline and follow-up, chi-square test modified by Mc Nemar was used. Categorical data were analyzed with Chi-square or Fisher test, respectively. The significance level was set at *p* < 0.05.

## 3. Results

### 3.1. Patients

A total of 88 participants completed the follow-up examination and were considered for analysis. The HC also contained 88 participants with comparable age, gender and smoking habits (Table 2). Thereby, 31 patients had received HTx, 43 had a LVAD and 14 patients suffered from HF. Within the HTx subgroup, a significantly higher amount of female patients was apparent (*p* < 0.01), while the LVAD group had the highest mean-age (*p* = 0.02). Smoking habits were comparable between subgroups (Table 2).

### 3.2. Oral Examination Baseline and Follow-up

Compared to the HC, the total cohort of patients with severe heart diseases suffered from a higher M-T (10.7 ± 9.0 vs. 3.2 ± 2.9, *p* < 0.01) and had less F-T (7.2 ± 5.2 vs. 14.8 ± 4.8, *p* < 0.01). Further oral conditions were comparable with HC (Table 3). Within the total cohort, D-T (0.4 ± 1.5 vs. 0.6 ± 1.1, *p* = 0.03) and M-T (10.7 ± 9.0 vs. 11.1 ± 9.2, *p* < 0.01) were slightly higher after 12 months. The periodontal treatment need could be significantly reduced from 91% at baseline to 75% at follow-up (after 12 months; *p* < 0.01). In the subgroups according to heart disease/condition, different changes could be observed. For the HTx subgroup, no significant differences of oral outcome parameters between baseline and follow-up were observed (*p* > 0.05). In the LVAD subgroup, slightly higher M-T (13.9 ± 8.8 vs. 14.4 ± 9.0, *p* < 0.01) and reduced periodontal treatment need (88% vs. 70%, *p* = 0.01) could be found at follow-up compared to baseline. Patients with HF only suffered from more missing teeth at the follow-up examination (9.8 ± 9.9 vs. 10.3 ± 9.9; *p* = 0.03). The comparison of baseline and follow-up is presented in Table 4.

### 3.3. Questionnaire

The majority of patients (79.5%) followed the recommendation to visit their dentist, without significant differences between subgroups (HTx, LVAD and HF). The most frequently applied measure was a (professional) tooth cleaning (53% of total cohort), whereby the LVAD group (40%) showed significantly lower number of participants who received tooth cleaning compared to HTx (68%) and HF (64%, *p* = 0.04). Despite of a baseline periodontal treatment need of 91%, only 10% of the total cohort received periodontal treatment during the 12 months observational period. Significantly more patients in the HTx subgroup rated a change in oral hygiene since heart disease (52% vs. 21% (LVAD) and 36% (HF), *p* = 0.03) and a higher importance of oral health since heart disease (61% vs. 26% (LVAD) and 21% (HF), *p* < 0.01). The detailed results of the questionnaire in the total cohort and subgroups are presented in Table 5.

### 3.4. Associations between Questionnaire and Follow-up Results

Different questions were analyzed regarding their potential association to the outcome in DMF-T, D-T, M-T, F-T as well as periodontal and dental treatment need at follow-up. Thereby, no statistical significant associations between oral health parameters and different questions were detected (*p* > 0.05). Table 6 presents the p-values for the respective analyses for the questions “Followed recommendation to dental visit”, “Dental visit because of complaints”, “Measures performed by dentist”, “Change in oral hygiene since heart disease” and “Higher importance of oral health since heart disease” (Table 6).

## 4. Discussion

Summary of the main results: The total cohort of patients with severe heart diseases had more missing and less filled teeth compared to the HC. The periodontal treatment need was reduced from 91% to 75% in the total cohort, whereby within the subgroups, only the LVAD patients showed a significant reduction in periodontal treatment need. The majority of participants followed the recommendation to visit the dentist. Despite of a periodontal treatment need of 91%, only 10% of the total cohort received periodontal treatment. The HTx subgroup reported more frequently changes in oral hygiene and higher importance of oral health since heart disease. No associations between the questionnaire-based assessment and oral health outcome were observed.

Comparison with published data: This is the first study that examined the effect of a standardized recommendation on the oral health as well as dental and periodontal treatment need of patients with HF, LVAD and HTx after a follow-up of 12 months. As there is no comparable study available, the interpretability considering published literature is limited.

Both oral findings at baseline and follow-up examination suggest that there would be a gap in dental care of these patients. Although periodontal treatment need was reduced during the follow-up period, it remains high with 75% in the total cohort. Approximately 75% of generally healthy individuals also suffer from periodontal treatment need, as presented in a recent representative study for the German general population [25]. Accordingly, there seems to be a lack in periodontal care in general. Similarly, the recruited HC with comparable age, gender and smoking habits suffered from a high periodontal treatment need. In contrast, caries prevalence and resulting dental treatment need was found to be approximately low and also comparable to HC and general population [25]. The higher number of missing teeth in the heart diseased cohort might indicate a surgical dental rehabilitation prior to HTx, LVAD implantation or listing for potential HTx in HF group. A previous cross-sectional study of patients with HF and after HTx also showed a high periodontal treatment need of these patients [18]. Moreover, another recent cross-sectional study of Chinese HTx recipients also found high periodontal treatment need for these patients [12]. Furthermore, patients with other solid organ transplantation, including kidney, liver and lung suffer from a high periodontal treatment need, regardless of their time period since transplantation [13]. Accordingly, the demand of an early rehabilitation and post-transplant maintenance [8], is not fulfilled, especially regarding periodontal burden. This is a serious issue, because periodontitis enhances the risk of oral bacteraemia, resulting in increased risk for infectious complications like endocarditis [26]. Insufficient dental and periodontal health in patients with heart diseases was also reported to be a risk factor for hospitalization and mortality [9]. However, improved oral health and care has the potential to positively influence the risk of cardiovascular events [10]. Therefore, treatment and prevention of periodontal inflammation is important to prevent (infectious) complications. Improved oral hygiene and reduced gingival inflammation can reduce transient bacteraemia during daily routine procedures like tooth brushing or chewing; additionally, therapeutic interventions like tooth extractions as a consequence of advanced periodontal burden might be avoided by improved long-standing preventive periodontal care [27]. While 79.5% of participants in the current study visited their dentist and 91% suffered from periodontal treatment need, only 10% received a periodontal treatment. Thereby, the periodontal treatment need outcome was not associated to periodontal treatment performed by dentist or dental visit in general. This encourages the assumption that the gap in periodontal care lies by the dentists, which are able to control dental caries, but not periodontal inflammation. However, this appears a more general issue, which might not exclusively be restricted to patients with HF or after HTx. The global burden of periodontal diseases is high, although actionable preventive, diagnostic and therapeutic strategies are available [28]. Accordingly, an insufficient periodontal care appears present, irrespective of the presence of a general disease. This might complicate an appropriate periodontal care of patients with severe general diseases like HF, LVAD and HTx. This could be additionally negatively influenced by the increased risk for complications of these patients during dental therapy [11,19,29,30], which might result in uncertainty of the dentist and thus in waiver of invasive (periodontal) therapy. This gap in periodontal treatment substantiates the demand of special care programs by specialized dentists with an interdisciplinary approach as demanded in literature [8,13,18].

Besides the gap in dental care, there seems to be several other conspicuities regarding the patient’s information and motivation. While the majority of participants followed the recommendation to visit their dentist, one fifth of the patients did not follow this recommendation. This is slightly worse than in the German general population where about 90% of healthy individuals visit their dentist at least once a year, whereby 92% visit the dentist control-oriented [25]. It has also been demonstrated, that patients with poorer general health show reduced utilization of dental services [31]. Accordingly, these current results appear in line with the available literature. Moreover, although HTx group scored better than LVAD and HF, only the minority of participants rated that their oral hygiene behavior had changed since heart disease. This points out to an assumption concluded from studies regarding oral health-related quality of life of organ transplant recipients: an inappropriate estimation of the importance of oral health caused by the general disease burden [32,33,34]. In heart transplanted individuals, the perceived influence of oral health on quality of life was concluded to be reduced [35]. Accordingly, an increased sensibilization and motivation for oral health issues, combined with the information about its importance for patient’s general health, seems necessary. There is still limited data on oral health promotion activities for patients with heart diseases [19]. However, improvements in periodontal as well as systemic health might be achieved by such interventions [19]. Therefore, this issue must be considered and included in an interdisciplinary special dental care program. Altogether, the fact that dental visit did not sufficiently improve the situation of patients would suggest an inadequate level of assistance for these patients by their dentists. This indicates the need for treating patients with severe heart disease in specialized centers, such as university hospitals, in collaboration with referring department within a special care concept.

Strengths and limitations: This is the first study that examined the potential effect of the recommendation for dental consultation on oral health as well as dental and periodontal treatment need in patients with HF, HTx and LVAD after 12 months follow-up. Accordingly, for the first time in literature, the potential reason for lack in oral care of these vulnerable patients has been demonstrated: The simple recommendation to visit the general private dental practitioner is not enough to reduce periodontal treatment need appropriately. At first glance, it seems trivial that visiting the dentist would lead to improved dental and periodontal conditions of the patients. The current study’s findings do not suggest that the dental visit is enough to sufficiently improve the situation. Therefore, it appears to be a clear hint that dental special care would be needed for these patients, because the current care system and concepts appear inappropriate. However, several limitations must be discussed. The recruitment of a large cohort of 88 participants is a strength, but a previous power calculation was not performed. Especially in the analysis of subgroups, the statistical power might be approximately too low to draw robust conclusions. The total cohort is thereby quite heterogeneous including patients with HF, LVAD and HTx, while different underlying diseases, co-morbidities and medication were not considered in the current study. Moreover, the high drop-out rate, whereby only about one quarter of patients completed the follow-up must be mentioned. This is a limitation and potential bias, because patients with low motivation and interest for oral health issues might be sorted out. Regarding clinical oral health parameters, the presence of missing teeth with the M-T according to WHO [22] must be considered. While this parameter regularly only considers missing teeth during caries, this has been modified in the current study. For further study, the presence of remaining teeth, especially considering remaining pairs of antagonists for occlusion, might be of higher clinical importance. Additionally, because every patient has received a doctor’s letter with the recommendation to visit their dentist, the effect of this measure cannot be finally assessed. For this, a control group without recommendation would have been needed, but due to ethical reasons, it was decided that every patient should receive the allocation to the dentist. In principle, the recommendation to visit the dentist cannot be properly considered as an “exposure”, because it is just a possibility of treatment. However, this reflects the recent clinical situation, where patients are allocated with a standardized doctor’s letter to receive dental control and therapy, if necessary. The result (dental consultation and/or therapy) has been evaluated for the first time for patients with severe heart diseases within this current study. This is of high practical relevance, because it is demonstrated that the recent concept just to allocate patients to private practitioners is not appropriate for this vulnerable group. In this context, the allocation to different private dental practitioners with a different state of qualification and/or specialization is a limitation of the current study, but it complies with the recent clinical situation. On the one hand, it is unclear whether these general practitioners are able and feel confident to apply the appropriate therapy. On the other hand, patients might have refused therapies due to economic concerns. Therefore, a specialized public dental unit might be more suitable. However, there is still no special care concept for these patients and the possibility to allocate patients to one specialized center could be limited by their right on free choice of doctor. An HC was composed based on an available patient pool for comparison of oral health conditions of the patients with severe heart diseases. Moreover, findings for the German, generally healthy population are available for interpretation [25]. The HC included patients, who regularly attend a dental clinic for routine control appointments. In comparison with the heart diseased individuals, this can be seen as potential bias and must be recognized in the interpretation of results. A further limitation is that the baseline and follow-up examination was performed by different dentists, what might explain minor differences between baseline and follow-up results. However, due to the application of standardized investigation procedures and calibration prior to examination, this effect might be small and not of high practical relevance. Furthermore, the dental behavior was just evaluated questionnaire-based from the patients’ perspective; thus, it is not clearly assessable whether patients really consulted their dentist and if the therapy they rated really has been performed. Considering these limitations, the driven conclusions should be interpreted carefully and the results must be seen as preliminary findings.

## 5. Conclusions

Within the limitations of the current study, it can be concluded that the simple recommendation to visit the dentist appears not enough to obtain sufficient dental and periodontal conditions in patients with severe heart diseases. There is a gap in dental care, especially periodontal treatment and maintenance of patients with HF, HTx and LVAD. Therefore, interdisciplinary care programs with dental specialists and the application of oral health promotion approaches are recommended.

## Figures and Tables

**Table 1:** Complexes of applied questionnaire and related content at the follow-up examination.

Complex	Content
Recommendation to dental visit	Was the recommendation to visit dentist followed or not Did the participants visit the dentist because of complaints during the last 12 months
Content of dental consultation	Performed measures by the dentist Information from the dentist about oral health and heart diseases
Antibiotic prophylaxis	Did the patients receive an antibiotic prophylaxis prior to dental therapy
Dentists knowledge and handling with the underlying disease	Dentists knowledge about underlying disease Regular update of health record and/or disease specific issues
Personal oral behavior in context of underlying disease	Changes in personal oral hygiene since diagnosis of heart disease Perceived higher importance of oral health since diagnosis of heart disease

**Table 2:** Patient characteristics, significance level: *p* < 0.05.

		Total Cohort (*n* = 88)	Healthy Compari- Son Group (*n* = 88)	*p*-value Total Cohort vs. Healthy Comparison	HTx (*n* = 31)	LVAD (*n* = 43)	HF (*n* = 14)	*p*-value Sub- Groups
Gender (female in % (n))		11% (10)	17% (15)	0.29	26% (8)	2% (1)	7% (1)	**<0.01**
Age in years (mv ± sd)		57.94 ± 10.88	58.17 ± 7.06	0.55	55.00 ± 9.24	60.44 ± 11.20	56.79 ± 12.09	**0.02**
Smo- king habits % (n)	Smo-ker	11% (10)	20% (15)	0.31	10% (3)	14% (6)	86% (12)	0.15
	non- smo-ker	76% (67)	64% (59)		87% (27)	65% (28)	7% (1)	
	For-mer smo-ker	13% (11)	16% (14)		3% (1)	21% (9)	7% (1)	

HTx: heart transplantation, LVAD: left-ventricular assist device, HF: heart failure, mv: mean value, sd: standard deviation, significant values are highlighted in bold.

**Table 3:** Oral conditions and treatment need between patients with heart diseases (total cohort including HF, HTx and LVAD at baseline) and the healthy comparison group.

Parameter	Total Cohort Heart Diseases (*n* = 88)	HC (*n* = 88)	*p*-value
D-T (mv ± sd)	0.4 ± 1.5	0.7 ± 1.24	0.37
M-T (mv ± sd)	10.7 ± 9.0	3.2 ± 2.9	**<0.01**
F-T (mv ± sd)	7.2 ± 5.2	14.8 ± 4.8	**<0.01**
DMF-T (mv ± sd)	18.3 ± 6.8	18.7 ± 5.2	0.56
dental treatment need %	16%	31%	0.19
periodontal treatment need %	91%	87%	0.69

mv: mean value, sd: standard deviation, D-T: number of decayed teeth, M-T: number of missing teeth, F-T: number of filled teeth, DMF-T: decayed-, missing- and filled-teeth index, significant values are highlighted in bold.

**Table 4:** Results of the dental examination in the total cohort and between groups at baseline and follow-up, significance level: *p* < 0.05.

	Total			HTx (*n* = 31)			LVAD (*n* = 43)			HF (*n* = 14)		
	Baseline	12 Months	*p*-value	Baseline	12 Months	*p*-value	Baseline	12 Months	*p*-value	Baseline	12 Months	*p*-value
D-T	0.4 ± 1.5	0.6 ± 1.1	**0.03**	0.4 ± 1.0	0.7 ± 1.2	0.12	0.6 ± 2.0	0.5 ± 1.1	0.43	0	0.4 ± 0.9	0.11
M-T	10.7 ± 9.0	11.1 ± 9.2	**<0.01**	6.7 ± 7.3	6.8 ± 7.5	0.32	13.9 ± 8.8	14.4 ± 9.0	**<0.01**	9.8 ± 9.9	10.3 ± 9.9	**0.03**
F-T	7.2 ± 5.2	7.2 ± 5.3	0.80	9.2 ± 5.5	9.4 ± 5.8	0.55	5.7 ± 4.4	5.7 ± 4.3	0.82	7.4 ± 5.3	7.1 ± 5.7	0.82
DMF-T	18.3 ± 6.8	18.8 ± 6.7	0.01	16.4 ± 6.7	16.8 ± 6.8	0.09	20.2 ± 6.1	20.7 ± 5.9	0.07	17.1 ± 7.7	17.8 ± 7.5	0.35
Dental treatment need	16%	25%	0.13	23%	29%	0.75	16%	23%	0.51	0%	21%	0.30
Periodontal treatment need	91%	75%	**<0.01**	97%	87%	0.25	88%	70%	**0.01**	86%	64%	0.25
Overall treatment need	91%	81%	**0.01**	97%	90%	0.50	88%	74%	**0.03**	86%	79%	0.99

HTx: heart transplantation, LVAD: left-ventricular assist device, HF: heart failure, mv: mean value, sd: standard deviation, D-T: number of decayed teeth, M-T: number of missing teeth, F-T: number of filled teeth, DMF-T: decayed-, missing- and filled-teeth index, significant values are highlighted in bold.

**Table 5:** Results of the questionnaire regarding dental consultation. Values are given as % (*n*) significance level: *p* < 0.05.

Parameter		Total	HTx	LVAD	HF	*p*-value
Followed recommendation to dental visit		79.5% (70/88)	87% (27/31)	74% (32/43)	79% (11/14)	0.41
Dental visit because of complaints		23% (20/88)	23% (7/31)	16% (7/43)	43% (6/14)	0.12
Measures performed by dentist	Tooth cleaning	53% (47/88)	68% (21/31)	40% (17/43)	64% (9/14)	**0.04**
	Periodontal treatment	10% (9/88)	16% (5/31)	5% (2/43)	14% (2/14)	0.24
	Restorative dentistry	14% (12/88)	26% (8/31)	7% (3/43)	7% (1/14)	0.05
	Tooth extraction	11% (10/88)	7% (2/31)	12% (5/43)	21% (3/14)	0.34
	Just control	15% (13/88)	10% (3/31)	19% (8/43)	14% (2/14)	0.57
How good were you informed from dentist about oral health and heart disease	Very good	15% (13/88)	26% (8/31)	9% (4/43)	7% (1/14)	0.19
	Sufficient	33% (29/88)	32% (10/31)	30% (13/43)	43% (6/14)	
	Only little	12 (11/88)	3% (1/31)	16% (7/43)	21% (3/14)	
	Not at all	40% (35/88)	39% (21/31)	44% (19/43)	29% (4/14)	
Received antibiotic prophylaxis		47% (36/76)	53% (16/30)	40% (15/38)	63% (5/8)	0.57
Dentists knowledge about underlying disease		90% (69/77)	93% (28/30)	87% (31/35)	83% (10/12)	0.61
Regular update of health record by dentist		73% (45/62)	70% (16/23)	71% (20/28)	82% (9/11)	0.74
Change in oral hygiene since heart disease		35% (30/87)	52% (16/31)	21% (9/42)	36% (5/14)	**0.03**
Higher importance of oral health since heart disease		38% (33/87)	61% (19/31)	26% (11/42)	21% (3/14)	**<0.01**

HTx: heart transplantation, LVAD: left-ventricular assist device, HF: heart failure, significant values are highlighted in bold.

**Table 6:** Association between questionnaire and oral health in the total cohort after 12 months, significance level: *p* < 0.05.

Parameter		DMF-T	D-T	M-T	F-T	Periodontal Treatment Need	Dental Treatment Need
Followed recommendation to dental visit		*p* = 0.72	*p* = 0.39	*p* = 0.25	*p* = 0.27	*p* = 0.54	*p* = 0.37
Dental visit because of complaints		*p* = 0.70	*p* = 0.36	*p* = 0.52	*p* = 0.54	*p* = 0.57	*p* = 0.57
Measures performed by dentist	Tooth cleaning	*p* = 0.64	*p* = 0.97	*p* = 0.96	*p* = 0.65	*p* = 0.46	*p* = 0.99
	Periodontal treatment	*p* = 0.51	*p* = 0.42	*p* = 0.80	*p* = 0.25	*p* = 0.69	*p* = 0.44
	Restorative dentistry	*p* = 0.44	*p* = 0.46	*p* = 0.36	*p* = 0.17	*p* = 0.08	*p* = 0.72
	Tooth extraction	*p* = 0.13	*p* = 0.93	*p* = 0.11	*p* = 0.40	*p* = 0.99	*p* = 0.71
	Just control	*p* = 0.27	*p* = 0.51	*p* = 0.92	*p* = 0.91	*p* = 0.08	*p* = 0.50
Change in oral hygiene since heart disease		*p* = 0.47	*p* = 0.74	*p* = 0.21	*p* = 0.41	*p* = 0.19	*p* = 0.80
Higher importance of oral health since heart disease		*p* = 0.07	*p* = 0.93	*p* = 0.09	*p* = 0.53	*p* = 0.13	*p* = 0.99

HTx: heart transplantation, LVAD: left-ventricular assist device, HF: heart failure, D-T: number of decayed teeth, M-T: number of missing teeth, F-T: number of filled teeth, DMF-T: decayed-, missing- and filled-teeth index, significant values are highlighted in bold.

## References

[1] Tzoulaki I., Elliott P., Kontis V., Ezzati M. (2016). Worldwide Exposures to Cardiovascular Risk Factors and Associated Health Effects: Current Knowledge and Data Gaps. Circulation.

[2] Bansilal S., Castellano J.M., Fuster V. (2015). Global burden of CVD: Focus on secondary prevention of cardiovascular disease. Int. J. Cardiol..

[3] Guha K., McDonagh T. (2013). Heart failure epidemiology: European perspective. Curr. Cardiol. Rev..

[4] Prinzing A., Herold U., Berkefeld A., Krane M., Lange R., Voss B. (2016). Left ventricular assist devices-current state and perspectives. J. Thorac. Dis..

[5] Lund L.H., Edwards L.B., Dipchand A.I., Goldfarb S., Kucheryavaya A.Y., Levvey B.J., Meiser B., Rossano J.W., Yusen R.D., Stehlik J. (2016). International Society for Heart and Lung Transplantation. The Registry of the International Society for Heart and Lung Transplantation: Thirty-third Adult Heart Transplantation Report-2016; Focus Theme: Primary Diagnostic Indications for Transplant. J. Heart Lung Transplant..

[6] Harms K.A., Bronny A.T. (1986). Cardiac transplantation: Dental considerations. J. Am. Dent. Assoc..

[7] Golder D.T., Drinnan A.J. (1993). Dental aspects of cardiac transplantation. Transplant. Proc..

[8] Rustemeyer J., Bremerich A. (2007). Necessity of surgical dental foci treatment prior to organ transplantation and heart valve replacement. Clin. Oral Investig..

[9] Joshy G., Arora M., Korda R.J., Chalmers J., Banks E. (2016). Is poor oral health a risk marker for incident cardiovascular disease hospitalisation and all-cause mortality? Findings from 172 630 participants from the prospective 45 and Up Study. BMJ Open.

[10] Park S.Y., Kim S.H., Kang S.H., Yoon C.H., Lee H.J., Yun P.Y., Youn T.J., Chae I.H. (2019). Improved oral hygiene care attenuates the cardiovascular risk of oral health disease: A population-based study from Korea. Eur. Heart J..

[11] Morimoto Y., Nakatani T., Yokoe C., Kudo C., Hanamoto H., Niwa H. (2015). Haemostatic management for oral surgery in patients supported with left ventricular assist device—A preliminary retrospective study. Br. J. Oral Maxillofac. Surg..

[12] Cao Y., Chen X., Jia Y., Lv Y., Sun Z. (2018). Oral health status of adult heart transplant recipients in China: A cross-sectional study. Medicine (Baltimore).

[13] Schmalz G., Wendorff H., Berisha L., Meisel A., Widmer F., Marcinkowski A., Teschler H., Sommerwerck U., Haak R., Kollmar O. (2018). Association between the time after transplantation and different immunosuppressive medications with dental and periodontal treatment need in patients after solid organ transplantation. Transpl. Infect. Dis..

[14] Ziebolz D., Hraský V., Goralczyk A., Hornecker E., Obed A., Mausberg R.F. (2011). Dental care and oral health in solid organ transplant recipients: A single center cross-sectional study and survey of German transplant centers. Transpl. Int..

[15] Schmalz G., Kauffels A., Kollmar O., Slotta J.E., Vasko R., Müller G.A., Haak R., Ziebolz D. (2016). Oral behavior, dental, periodontal and microbiological findings in patients undergoing hemodialysis and after kidney transplantation. BMC Oral Health.

[16] Kauffels A., Schmalz G., Kollmar O., Slotta J.E., Weig M., Groß U., Bader O., Ziebolz D. (2017). Oral findings and dental behaviour before and after liver transplantation—A single-centre cross-sectional study. Int. Dent. J..

[17] Marcinkowski A., Ziebolz D., Kleibrink B.E., Weinreich G., Kamler M., Teschler H., Sommerwerck U. (2018). Deficits in oral health behavior and oral health status in patients after lung transplantation. Clin. Respir. J..

[18] Binner C., Wagner J., Schmalz G., Eisner M., Rast J., Kottmann T., Haak R., Oberbach A., Borger M.A., Garbade J. (2019). Insufficient Oral Behaviour and the High Need for Periodontal Treatment in Patients with Heart Insufficiency and after Heart Transplantation: A Need for Special Care Programs?. J. Clin. Med..

[19] Lam O.L., Zhang W., Samaranayake L.P., Li L.S., McGrath C. (2011). A systematic review of the effectiveness of oral health promotion activities among patients with cardiovascular disease. Int. J. Cardiol..

[20] Ziebolz D., Reiss L., Schmalz G., Krause F., Haak R., Mausberg R.F. (2018). Different views of dentists and general medical practitioners on dental care for patients with diabetes mellitus and coronary heart diseases: Results of a questionnaire-based survey in a district of Germany. Int. Dent. J..

[21] Wilson W., Taubert K.A., Gewitz M., Lockhart P.B., Baddour L.M., Levison M., Bolger A., Cabell C.H., Takahashi M., Baltimore R.S. (2007). Prevention of infective endocarditis: Guidelines from the American Heart Association: A guideline from the American Heart Association Rheumatic Fever, Endocarditis, and Kawasaki Disease Committee, Council on Cardiovascular Disease in the Young, and the Council on Clinical Cardiology, Council on Cardiovascular Surgery and Anesthesia, and the Quality of Care and Outcomes Research Interdisciplinary Working Group. Circulation.

[22] WHO (1997). Oral Health Surveys, Basic Methods.

[23] Diamanti-Kipioti A., Papapanou T.N., Moraitaki-Zamitsai A., Lindhe J., Mitsis F. (1993). Comparative estimation of periodontal conditions by means of different index systems. J. Clin. Periodontol..

[24] Meyle J., Jepsen S. (2000). The Periodontal Screening-Index (PSI). Parodontology.

[25] Jordan R.A., Micheelis W. (2016). The Fifth German Oral Health Study (DMS V). Institut der Deutschen Zahnärzte (Hrsg.).

[26] Dhotre S., Jahagirdar V., Suryawanshi N., Davane M., Patil R., Nagoba B. (2018). Assessment of periodontitis and its role in viridans streptococcal bacteremia and infective endocarditis. Indian Heart J..

[27] Carinci F., Martinelli M., Contaldo M., Santoro R., Pezzetti F., Lauritano D., Candotto V., Mucchi D., Palmieri A., Tagliabue A. (2018). Focus on periodontal disease and development of endocarditis. J. Biol. Regul. Homeost. Agents.

[28] Tonetti M.S., Jepsen S., Jin L., Otomo-Corgel J. (2017). Impact of the global burden of periodontal diseases on health, nutrition and wellbeing of mankind: A call for global action. J. Clin. Periodontol..

[29] Herman W.W., Ferguson H.W. (2010). Dental care for patients with heart failure: An update. J. Am. Dent. Assoc..

[30] Nunn P. (2000). Medical emergencies in the oral health care setting. J. Dent. Hyg..

[31] Reda S.M., Krois J., Reda S.F., Thomson W.M., Schwendicke F. (2018). The impact of demographic, health-related and social factors on dental services utilization: Systematic review and meta-analysis. J. Dent..

[32] Schmalz G., Kollmar O., Vasko R., Müller G.A., Haak R., Ziebolz D. (2016). Oral health-related quality of life in patients on chronic haemodialysis and after kidney transplantation. Oral Dis..

[33] Schmalz G., Wendorff H., Marcinkowski A., Weinreich G., Teschler H., Haak R., Sommerwerck U., Ziebolz D. (2018). Oral health related quality of life depending on oral health and specific factors in patients after lung transplantation. Clin. Respir. J..

[34] Schmalz G., Meisel A., Kollmar O., Kauffels A., Slotta J.E., Kottmann T., Haak R., Ziebolz D. (2018). Oral health-related quality of life depending on dental and periodontal health in different patients before and after liver transplantation. Clin. Oral. Investig..

[35] Segura-Saint-Gerons R., Segura-Saint-Gerons C., Alcántara-Luque R., Arizón-del Prado J.M., Foronda-Garcia-Hidalgo C., Blanco-Hungría A. (2012). Perceived influence of oral health upon quality of life in heart transplant patients. Med. Oral Patol. Oral Cir. Bucal.

